# Characterization of Flavonoids in the Ethomedicine Fordiae Cauliflorae Radix and Its Adulterant Millettiae Pulchrae Radix by HPLC-DAD-ESI-IT-TOF-MS*^n^*

**DOI:** 10.3390/molecules181215134

**Published:** 2013-12-09

**Authors:** Lan-Lan Fan, Tao Yi, Feng Xu, Ya-Zhou Zhang, Jian-Ye Zhang, Dian-Peng Li, Yang-Jiao Xie, Shan-Ding Qin, Hu-Biao Chen

**Affiliations:** 1 School of Chinese Medicine, Hong Kong Baptist University, Kowloon Tong, Hong Kong, China; 2 Guangxi Botanical Garden of Medicinal Plant, Nanning, Guangxi 530023, China; 3 School of Pharmaceutical Sciences, Peking University, Beijing 100191, China; 4 Guangxi Institute of Botany, the Chinese Academy of Science, Guilin, Guangxi 541006, China; 5 School of Pharmacy, Guangxi University of Chinese Medicine, Guangxi 530001, China

**Keywords:** *Fordia cauliflora*, *Millettia pulchra* var. *laxior*, HPLC-DAD-ESI-IT-TOF-MS^n^, flavonoids, identification, ethnomedicine

## Abstract

Fordiae Cauliflorae Radix (FC, the root of *Fordia cauliflora* Hemsl) and Millettiae Pulchrae Radix [MP, the root of *Millettia pulchra* (Benth.) Kurz var. *laxior* (Dunn) Z. Wei], which go under the same local name of “Daluosan”, have long been used in Southern China for the treatment of stroke, paralysis, dementia in children, Alzheimer’s disease and other diseases*.* The same local name and similar functions always confuse users. To further utilize these two ethnodrugs and identify them unambiguously, an HPLC-DAD-ESI-IT-TOF-MS^n^ method was developed to separate and characterize the flavonoids in FC and MP. A total of 41 flavonoids were detected, of which six compounds were identified by comparing their retention time and MS data with those of the reference standards, and the others were tentatively identified based on their tandem mass spectrometry data obtained in the positive ion detection mode. Nineteen of these characterized compounds are reported from these two plants for the first time.

## 1. Introduction

**Figure 1:**
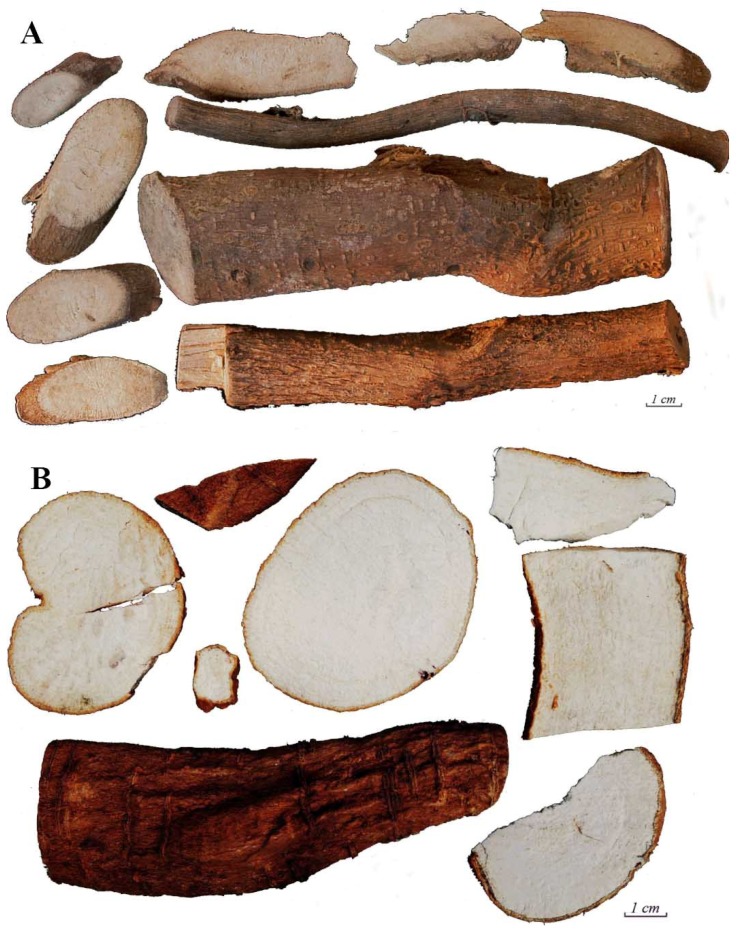
Photographs of Fordiae Cauliflorae Radix (**A**) and Millettiae Pulchrae Radix (**B**).

Fordiae Cauliflorae Radix, the Yao medicine from Guangxi Province of China, also named Daluosan, Shuiluosan, Tugancao or Xiaxudou, originates from the root of the Leguminosae family member *Fordia cauliflora* Hemsl (Figure 1A) [1,2,3]. It has been used for the treatment of stroke, paralysis, dementia in children, Alzheimer’s disease, traumatic brain injury and recovery of parturients for over five hundred years. Pharmacological studies have shown that its ethanol extract can improve learning and memory ability and reverse acquired memory disorder in mice [4,5], and that it has anti-aging [6], anti-inflammatory [7], hepatoprotective [8] and antioxidative [8] effects. Phytochemical studies have showed that the major constituents of FC are flavanoids, and the common chemical types are furanoflavones and pyranoflavones [2,9,10]. 

However, the local people often confuse FC with another Yao medicine, Millettiae Pulchrae Radix (MP), the root of *Millettia pulchra* (Benth.) Kurz var. *laxior* (Dunn) Z. Wei, which is also named Daluosan, Yulangsan, or Longyansen (Figure 1B) [3]. It was first recorded in the *Guangxi Herb Journal* [9], and expresses similar activities as FC, such as for the treatment of children with infantile malnutrition, or activating blood circulation to dissipate blood stasis [3,10]. Recent studies showed that MP had a wide range of biological cardiovascular system activities [11]. It also has nootropic effects [12], is able to protect the liver [13] and has the ability to scavenge oxygen free radicals [14]. 

As we know, naturally derived products play an important role as a source of medicines. Ethno-medicine development is a hotspot of global drug development. The reported literatures and folk usages of FC and MP indicated that these two Yao medicines have great potential in the treatment of cardiocerebral, vascular and nervous system diseases. However, the confusion of the two medicines and the lack of reports on the global analysis of their chemical constituents, hinder the further development of both medicines. We previously quantified five flavonoids in 15 *F. cauliflora* samples, including root, stem and leaves, and two root samples of *M. pulchra* var. *laxior*, and tried to compare the UPLC fingerprints of the two medicines [15]. Results showed that the UPLC fingerprints of FC and MP were quite consistent within species, but distinct from each other. However, more information should be provided. For further development of FC and MP there is an urgent need to elucidate the chemical constituents of these two plants. In this paper, we choose representative samples of FC and MP, and set up a HPLC-DAD-ESI-IT-TOF-MS^n^ method to illustrate their chemical characteristic details.

## 2. Results and Discussion

### 2.1. Optimization of HPLC Conditions

**Figure 2:**
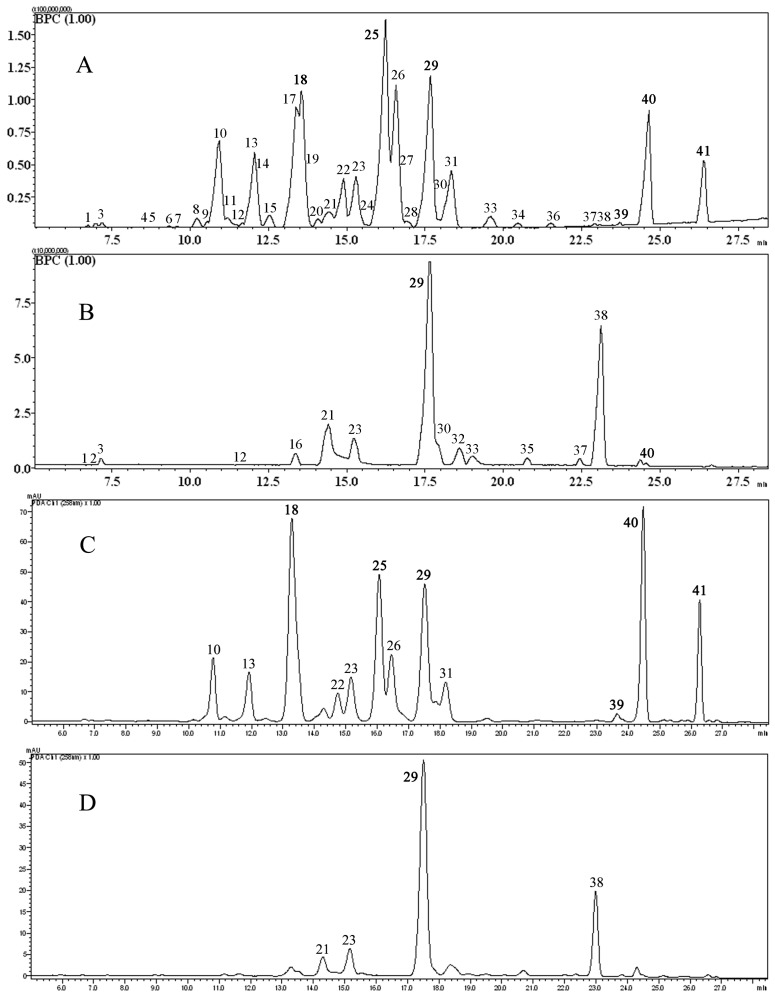
Base peak chromatograms (BPC) of (**A**) Fordiae Cauliflorae Radix and (**B**) Millettiae Pulchrae Radix, HPLC chromatograms of (**C**) Fordiae Cauliflorae Radix and (**D**) Millettiae Pulchrae Radix. Numbered compounds correspond to: **18**, *m/z* 353.1071, pachycarin A; **25**, *m/z* 323.0909, 3',4'-dimethoxy [2",3":7,8]furanoflavone; **29**, *m/z* 293.0796, karanjin; **39**, *m/z* 279.0641, pongaglabol; **40**, *m/z* 335.1270, karanjachromene and **41**, *m/z* 323.1617, isoderricin A.

In order to obtain desirable HPLC chromatograms, the procedure of sample preparation was optimized in terms of the extraction solvent, extraction times of flavonoids. Four different solvents, including methanol, 80% methanol, 50% methanol and ethanol, were selected as the extraction solvents. Methanol produced the highest yield for most constituents, so it was applied as the final extraction solvent. Different columns (Merck Purospher® Star RP_18_, Agela Venusil ASB C_18_, and Dionex Acclaim® PolarAdvantage II C_18_) were tested for the separation of the sample. By comparison, the Dionex Acclaim® PolarAdvantage II C_18_ gave the best chromatographic resolution among the three columns. For the mobile phase, 0.1% (v/v) formic acid was added to improve the mass spectrometry ionization efficiency and enable symmetric peak shapes. The detection wavelength was set at 258 nm, at which most flavonoid components can be detected with greatest sensitivity. The HPLC PDA chromatograms and LC/MS base peak chromatograms (BPC) of FC and MP are given in Figure 2.

### 2.2. Optimization of Mass Spectrometry Conditions

Both the positive and negative ion modes were tested for the reference flavonoids. Since during our study, MS and MS^n^ fragmentions gave more information in positive ion mode, analysis was therefore conducted in positive ion mode. 

### 2.3. Rationale for the Characterization of Flavonoids

Known compounds in the herbal extract were identified by comparing with reference compounds according to the retention time and MS^n^ spectra. Six peaks were identified by comparing with reference standards as pachycarin A (**18**), 3',4'-dimethoxy[2",3":7,8]furanoflavone (**25**), karanjin (**29**), pongaglabol (**39**), karanjachromene (**40**) and isoderricin A (**41**). All reference compounds exhibited [M+H]^+^ ions of sufficient abundance in MS. The MS^n^ spectra obtained from the reference compounds allowed us to propose the possible schemes for the fragmentation pathways of furanoflavones and pyranoflavones, and this information was used to elucidate the structure of unknown compounds. 

**Figure 3:**
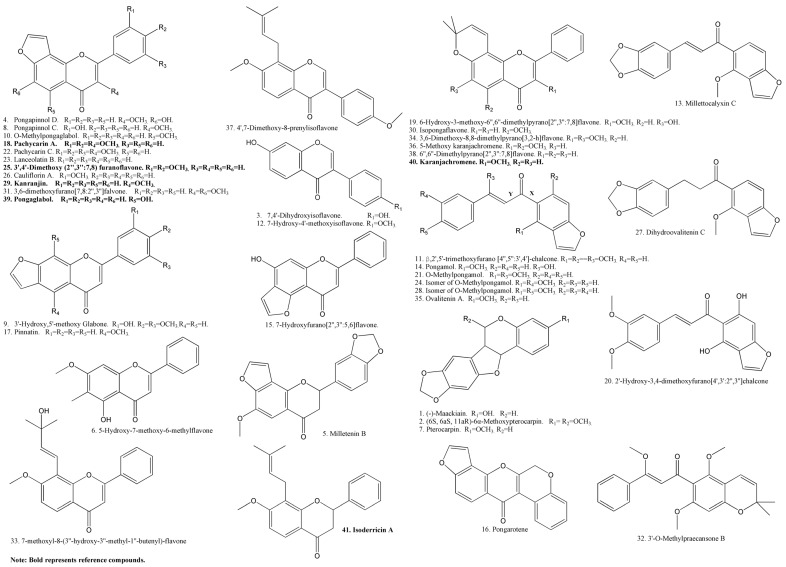
Structures of reference compounds and identified compounds.

A database (the Supplementary information-Table S1) was set up according to the reported chemicals isolated from *F. cauliflora* and *M. pulchra* var. *laxior*, including chemical names, structures, molecular formulae, molecular weights and so on. The elucidation procedure of unknown compounds was as follows: first of all, the molecular formulae of unknown compounds were calculated from their HRMS data, and the characteristic fragments of them were also summarized, and then the information was compared with the database. If the molecular formulae and the major fragment ions of certain compounds matched the reported chemicals in the database, their structures were elucidated. However, if the molecular formulae could not be matched with any chemicals in the database, or the molecular formulae could be matched with the database but the major fragment ions could not be matched, then they will be compared with the data retrieved in SciFinder, Dictionary of Natural Products and so on. The most plausible structure was elucidated through comprehensive analysis of MS^n^ data. The UV spectra of the chemicals were also used to judge their structures. Meanwhile, the characteristic neutral losses of 16 Da (CH_4_), 18 Da (H_2_O), 28 Da (CO), 29 Da (HCO), 31 Da (CH_4_+CH_3_), 33 Da (H_2_O+CH_3_), 43 Da (CO+CH_3_), 44 Da (CO_2_), 46 Da (H_2_O+CO), and 61 Da (CO+H_2_O+CH_3_) were also frequently observed in their MS^2^ and MS^3^ spectra. A total of 41 flavonoids were characterized (Table 1). Nineteen compounds were reported from FC and MP for the first time. The tentatively identified structures and compound names are shown in Figure 3. 

#### 2.3.1. Identification of Furonoflavonoids

**Figure 4:**
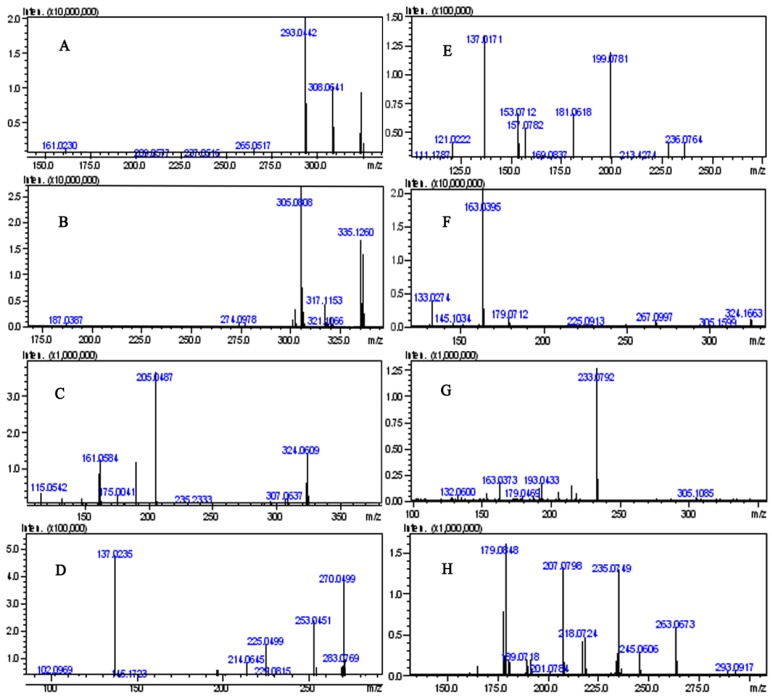
ESI-MS/MS of selected ions from the chromatograms presented in Figure 2: (**A**) *m/z* 323.0909, compound **25**, 3',4'-dimethoxy(2",3":7,8)furanoflavone; (**B**) *m/z* 335.1270, compound **40**, karanjachromene; (**C**) *m/z* 339.1128, compound **11**, *β*,2',5'-trimethoxyfurano [4'',5'':3',4']-chalcone; (**D**) *m/z* 285.0755, compound **1**, (−)-maackiain; (**E**) *m/z* 255.0642, compound **3**, 7,4'-dihydroxyisoflavone; (**F**) *m/z* 323.1617, compound **41**, isoderricin A; (**G**) *m/z* 337.1428, compound **33**, 7-methoxyl-8-(3''-hydroxy-3''-methyl-1''-butenyl)-flavone; (**H**) *m/z* 291.0649, compound **16**, pongarotene.

**Figure 5:**
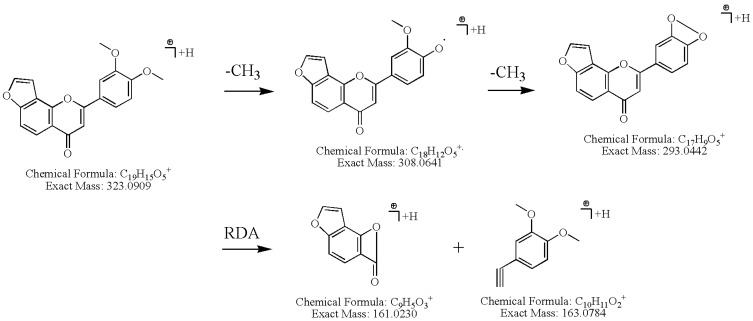
Proposed fragmentation pathways for compound **25**, 3',4'-dimethoxy(2'',3'': 7,8)-furanoflavone.

The pseudo-molecule ion [M+H]^+^ of 3',4'-dimethoxy(2",3":7,8)furanoflavone, peak **25**, in the positive ion mode was *m/z* 323.0909, indicating that its molecular formula was C_19_H_1__4_O_5_ (Table 1). It loses one and two methyl radicals (CH_3_) in its MS^2^ spectra, and formed the base peaks of [M+H–15]^+^ and [M+H–30]^+^ at *m/z* 308.0641 (C_1__8_H_1__2_O_5_) and 293.0442 (C_1__7_H_9_O_5_), respectively. It also further generated the characteristic ions at *m/z* 161.0230 (C_9_H_5_O_3_) and 163.0784 (C_10_H_11_O_2_) in its MS^2^ spectrum (Figure 4A). It could be deduced that the dominating fragmentation pathway was retro-Diels-Alder (RDA) cleavage from the 1,3-position of the C-ring. And the ^1,3^A^+^ ion, *m/z* 161.02 (C_9_H_5_O_3_) was the characteristic fragment ion of furanoflavone. The proposed fragmentation pathway can be seen in Figure 5.

The molecular formulae of compounds **10**, **17**, **26** and **29** were determined to be C_18_H_12_O_4_ according to their HRMS data (Table 1). Compound **29** was identified as karanjin by reference [16]. Karanjin contained a 3-methoxyl moiety, and it loses a CH_3_ and CH_4_ in its MS^2^ spectrum. In the PI MS^2^ spectra of both compounds **10** and **17**, the characteristic fragment ions at *m/z* 278.06 (predicted to be C_1__7_H_10_O_4_) and *m/z* 176.01 (C_9_H_5_O_4_, ^1,3^A^+^) formed by RDA clearage suggested that the methoxyl group was link to the A-ring. According to the reported chemicals from the FC, compounds **10** and **17** were tentatively identified as *O*-methylpongaglabol [17] and pinnatin [18], respectively. By contrast, the fragment ions at *m/z* 250.06 (C_16_H_10_O_3_, [M+H-CH_3_-CO]^+^, base peak) and 161.02 (C_9_H_5_O_3_, ^1,3^A^+^) were observed in the MS^2^ spectrum of compound **26**, indicating that the methoxyl group was linked to the B-ring. Therefore, **26** was tentatively identified as cauliflorin A according to the reported literature [18].

The predicted molecular formulae of compounds **4** and **8** were C_18_H_12_O_5_ based on their HRMS data (Table 1). In their MS^2^ spectra, the characteristic ions at *m/z* 294.05 (C_1__7_H_10_O_5_, [M+H-CH_3_]^+^) and *m/z* 176.01 (C_9_H_4_O_4_, ^1,3^A^+^) were detected in compound **4**, while the characteristic ions at *m/z* 294.05 (C_1__7_H_10_O_5_, [M+H-CH_3_]^+^), *m/z* 266.06 (C_1__6_H_9_O_4_, [M+H-CH_3_-CO]^+^) and *m/z* 161.02 (C_9_H_5_O_3_, ^1,3^A^+^) were detected in compound **8**. Therefore, the hydroxyl group was linked to the A-ring of compound **4**, while for compound **8** was B-ring or C-ring. So they were tentatively identified as pongapinnol D [19] and pongapinnol C by comparing with the reported chemicals in the *Millettia* genus [18], respectively.

The molecular formulae of compounds **15** and **39** were determined to be C_17_H_10_O_4_ according to their HRMS data (Table 1). Compound **39** was identified as pongaglabol by comparing with the reference compound [15]. In its PI MS^2^ spectrum, the fragment ions at *m/z* 149.02 (C_8_H_5_O_3_, ^1,4^A^+^) and *m/z* 177.01 (C_9_H_5_O_4_, ^1,3^A^+^) were observed. However, the fragment ions at *m/z* 149.02 (C_8_H_5_O_3_, ^1,4^A^+^) and *m/z* 176.01 (C_9_H_4_O_4_, ^1,3^A^+^) were also detected in compound **15**, which means that its hydroxy group was link to the A-ring. So compound **15** was tentatively identified as 7-hydroxyfurano[2'',3'':5,6]flavone by comparing with the literature [20].

#### 2.3.2. Identification of Pyranoflavonoids

Compound **40** was identified as karanjachromene, a pyranoflavone, by the reference [15]. The molecular formula was calculated to be C_21_H_18_O_4_ based on HRMS data (Table 1). In the MS^2^ spectrum (Figure 4B), the fragment ions at *m/z* 317.1153 (C_21_H_17_O_3,_ [M-H_2_O]^+^), *m/z* 305.0808 (C_19_H_13_O_4,_ [M-2CH_3_]^+^), and *m/z* 187.0387 (C_11_H_7_O_3_, ^1,4^A^+^) were observed. So the characteristic ions at *m/z* 187.04 (C_11_H_7_O_3_, ^1,4^A^+^) or its hydroxyl substitute ions can be treated as the judgment of pyranoflavnoids.

The molecular formula of compound **19** was determined to be C_21_H_18_O_5_ according to its HRMS data (Table 1). In its MS^2^ spectrum, *m/z* 321.0751 (C_19_H_1__3_O_5_, [M-2CH_3_]^+^), *m/z* 279.0685 (C_17_H_1__1_O_4_, [M-2CH_3_-CH_2_-CO]^+^) and *m/z* 205.07 (C11H9O4, ^1,4^A^+^) were observed. According to the report, compound **19** was tentatively identified as 6-hydroxy-3-methoxy-6'',6''-dimethylpyrano[2'',3'':7,8]flavone [21]. 

#### 2.3.3. Identification of Chalcones

The molecular formula of compound **11** was determined to be C_20_H_18_O_5_ according to its HRMS data (Table 1). The RDA cleavage of it at bond Y to yield the base peak ion ^Y^A^+^ at *m/z* 205.0487 (elemental composition: C_11_H_9_O_4_) and at bond X to yield the minor ion ^X^B^+^ at *m/z* 161.0584 (elemental composition: C_10_H_9_O_2_) could also be simultaneously detected in the MS^2^ spectrum. The ^Y^A^+^ fragment also loses one and two methyl radicals (CH_3_) in its MS^2^ spectra (Figure 4C) means that the A ring contains two methoxy moieties [22]. The fragmentation pathway was highly similar with what happened to flavanones. This is reasonable because cyclization of 6'-hydroxychalcones to flavanones has been reported in a number of studies demonstrating the presence of an intramolecular equilibrium between a flavanone-type and a chalcone-type molecular ion [23]. 

#### 2.3.4. Identification of Pterocarpin, Isoflavone, Flavones, Flavonones and Rotenoids

Compounds **1**, **2** and **7** were pterocarpans. Referring to compound **1** for example, its predicted formula was C_16_H_12_O_5_ (Table 1), the RDA cleavage fragment ion at *m/z* 137.0235 (elemental composition: C_7_H_5_O_3_) was observed (Figure 4D) and in accordance with its structure [24].

Compounds **3** and **12** were isoflavones. The predicted molecular formulae of compounds **3** and **12** were C_15_H_10_O_4_ and C_16_H_12_O_4_, respectively, based on their HRMS data (Table 1). The characteristic fragment ion at *m/z* 137.02 (C_7_H_5_O_3_, ^1,3^A^+^), which was produced after RDA cleavage from the 1,3-position of the C-ring, both existed in their MS^2^ spectra (Figure 4E) [21].

Compounds **5** and **41** were flavonones. Compound **41** was identified by comparing with the reference compound (Figure 3) and literature [15], its MS^2^ spectrum is showed in Figure 4F. Compound **5** lost a CH_4_ in its MS^2^ spectrum, and no characteristic ion at *m/z* 161.02 (C_9_H_5_O_3_, ^1,3^A^+^) was detected, so it is not a flavone, and it was identified as milletenin B according to the literature [25], a flavonone. Compounds **6** and **33** were tentatively identified as 5-hydroxy-7-methoxy-6-methylflavone [26] and 7-methoxyl-8-(3''-hydroxy-3''-methyl-1''-butenyl)-flavone [27], respectively. The MS^2^ spectrum of compound **33** is given in Figure 4G.

Compound **16** was a rotenoid. The major fragment ions in its MS^2^ spectrum (Figure 4H) resulted from losing 28 Da (CO) and 18 Da (H_2_O) [28].

#### 2.3.5. Chemical Characteristics of FC and MP

The base peak chromatograms (BPC) and PDA chromatograms of FC and MP are shown in Figure 2. In total, 41 flavanoids, including two isoflavones (two known), three pterocarpans (three known), one rotenoid, 10 chalcones (two known), 14 furanoflavones (nine known), seven pyranoflavones (four known), two flavones (one known), and two flavonones (one known) were tentatively identified, and the peak area of each compound calculated from their extracted ion chromatograms (EICs) was shown in Table 1. This is the first report of 19 chemicals from the two ethnomedicines. Some peaks were too weak to be seen clearly in the base peak chromatograms (BPCs).

Among the 41 peaks, 37 peaks were detected in FC, including 14 furanoflavones, seven pyranoflavones, eight chalcones, two isoflavones, two flavones, two flavonones and two pterocapans. Furanoflavones, pyranoflavones and chalcones are the major chemical types. However, only 15 peaks were detected in MP, including two furanoflavones, four pyranoflavones, three chalcones, two isoflavones, one rotenoid, one flavone and two pterocapans. Thus, furanoflavones were the major flavonoid chemical types in FC, while for MP the major types were chalcones and pyranoflavones. There are 11 common peaks, which are (−)-maackiain (**1**), 7,4'-dihydroxyisoflavone (**3**), 7-hydroxy-4'-methoxyisoflavone (**12**), 7-hydroxy-4'-methoxyisoflavone (**21**), lanceolatin B (**23**), karanjin (**29**), isopongaflavone (**30**), 7-methoxyl-8-(3''-hydroxy-3''-methyl-1''-butenyl)-flavone (**33**), 4',7-dimethoxy-8-prenylisoflavone (**37**), 6'',6''-dimethylpyrano[2'',3'':7,8]flavone (**38**) and karanjachromene (**40**), between FC and MP.

#### 2.3.6. Identification of FC and MP

Our previous report showed that karanjin (**29**) was the major common peak between FC and MP, and pachycarin A (**18**), 3',4'-dimethoxy[2",3":7,8]furanoflavone (**25**), karanjachromene (**40**) and isoderricin A (**41**) can be used to differentiate between FC and MP samples [15]. However, this study indicated there were 26 compounds which were detected in FC and were not in MP, and there were four chemicals that existed in MP but not in FC (Table 1 and Figure 2C). According to the detected area of each compound (Table 1), we suggested the characteristic chemicals detected in FC, whose peak area were higher than 10^7^, including *O*-methylpongaglabol (**10**), millettocalyxin C (**13**), pongamol (**14**), pinnatin (**17**), pachycarin A (**18**), 6-hydroxy-3-methoxy-6'',6''-dimethyl-pyrano[2'',3'':7,8]flavone (**19**), pachycarin C (**22**), 3',4'-dimethoxy[2",3":7,8]furanoflavone (**25**), cauliflorin A (**26**), 3,6-dimethoxyfurano[7,8:2'',3'']falvone (**31**) and isoderricin A (**41**), could be used to differentiate FC from MP.

However, the peak area ratio of karanjachromene (**40**) calculated from extracted ion chromatograms (EICs) of FC and MP was 103:1, so maybe this is the reason why karanjachromene was not detected in MP by ultra-performance liquid chromatography (UPLC) with triple-quadrupole mass spectrometry (QqQ-MS). 

Besides karanjin, karanjachromene was found to possess significant antioxidant activity [29]. Few pharmacological activities were reported for pachycarin A, 3',4'-dimethoxy[2",3": 7,8]furanoflavone and isoderricin A, even though they have been known for years. However, previous researchers showed that furanoflavones can be used as antibrowning agents [30] and can also have antioxidant and radical quenching activities [31]. Pyranoflavones have antimycobacterial [32] and cytotoxic activities [33], and so on. Such information can give us clues that furanoflavones and pyranoflavones play very important roles in FC and MP. Nowadays, flavonoids are famous for their various medical efficacies, such as cardioprotective effects, antithrombotic and vasoprotective effects, antioxidation and anti-aging activies, anti-inflammatory activities [29]. Thus, the therapeutic functions of FC and MP as treatment for stroke, dementia in children and Alzheimer’s disease may be due to their richness in flavonoids, but more experiments will need to be performed in order to prove this.

**Table 1:** Characterization of compounds detected in Fordiae Cauliflorae Radix (FC) and Millettiae Pulchrae Radix (MP) extract by HPLC-DAD-ESI-IT-TOF-MS^n^.

No.	t *_R_* (min)	Formula	PI meas. (Da)	PI pred. (Da)	Error (ppm)	Major Fragments Ions (PI)	Identification	Peak Area in FC	Peak Area in MP
1 ^b^	6.63	C_16_H_12_O_5_	285.0755	285.0685	0.9	**270.0499**,253.0451, 225.0499, 214.0645, **137.0235**	(−)-Maackiain [24]	303223	186752
2 ^b^	6.88	C_18_H_16_O_6_	329.1038	329.1020	5.5	314.0747, 299.0560, 191.0764, 167.0669, **147.0428**	(6S,6aS,11aR)-6α-Methoxy-pterocarpin [24]	-	691251
3 ^a^	7.46	C_15_H_10_O_4_	255.0642	255.0652	−1	236.0764, 199.0781, 181.0618, 153.0712, **137.0171**, 121.0222	7,4'-Dihydroxyisoflavone [21]	1124746	148674
4	8.64	C_18_H_12_O_5_	309.0756	309.0758	−0.2	**294.0495**, 238.0635, 192.0049,**176.0118**, 164.0106	Pongapinnol D [34]	760606	-
5	8.72	C_19_H_14_O_6_	339.0844	339.0863	−1.9	324.0552, **323.0544**, 321.0394, 295.0583, **293.0442**, 278.0568,181.0639	Milletenin B [25]	1482047	-
6	9.27	C_17_H_14_O_4_	283.0948	283.0956	−1.7	267.0627, 239.0704, 137.0277	5-Hydroxy-7-methoxy-6-methylflavone [26]	630180	-
7 ^b^	9.312	C_17_H_14_O_5_	299.0998	299.0914	−3.0	**284.0681**, 257.8259, 174.0634	Pterocarpin [24]	1885294	-
8 ^a^	10.26	C_18_H_12_O_5_	309.0749	309.0758	−0.9	**294.0496**, 266.0575, 210.0791, **161.0244**	Pongapinnol C [18]	7681020	-
9	10.59	C_19_H_14_O_6_	339.0848	339.0863	−1.5	324.0619, **309.0375**, 281.0413, 279.0628, 179.0845, **161.0245**	3'-Hydroxy,4',5'-dimethoxy furano[2'',3'':7,6]flavone [35]	5349406	-
10 ^a^	10.98	C_18_H_12_O_4_	293.0801	293.0808	−0.7	**278.0560**, 250.0590, 222.0635, 194.0715, **176.0103**, **148.0139**	*O*-Methylpongaglabol [17]	60114615	-
11 ^a^	11.32	C_20_H_18_O_5_	339.1128	339.1074	5.4	324.0609, **205.0487**, 190.0239, 175.0041, **161.0584**	β,2',5'-Trimethoxyfurano-[4'',5'':3',4']chalcone [22]	8167359	-
12 ^a^	11.57	C_16_H_12_O_4_	269.0800	269.0736	3.1	**254.0576**, 253.0494, **237.0542**, 209.0716, **181.0674**, 137.0234	7-Hydroxy-4'-methoxyisoflavone [21]	4302864	828786
13	12.11	C_19_H_14_O_5_	323.0899	323.0914	−1.5	**307.0601**, 305.0447, 279.0641, **277.0481**, 261.0529, **161.0215**, 145.0280	Millettocalyxin C [36]	57681679	-
14 ^b^	12.22	C_18_H_14_O_4_	295.0929	295.0965	−3.6	279.0843, 267.4780, **191.0352**, **176.0113**	Pongamol [23]	61222600	-
15	12.28	C_17_H_10_O_4_	279.0670	279.0652	1.8	205.0783, **176.0095**, **149.0231**	7-Hydroxyfurano[2'',3'':5,6]flavone [20]	4101706	-
16	13.14	C_18_H_10_O_4_	291.0649	291.0652	−0.3	263.0673, 235.0749, 217.0628, **207.0798**, **179.0848**	Pongarotene [28]	-	5862765
17 ^a^	13.37	C_18_H_12_O_4_	293.0794	293.0808	−1.4	**278.0559**, 250.0599, 222.0630, 194.0718, **176.0100**, **148.0157**	Pinnatin [18]	76746797	-
18 ^a^	13.65	C_20_H_16_O_6_	353.1071	353.1020	5.1	**338.0765**, 323.0557, **295.0576**, 277.0497, **161.0209**, 145.0294	Pachycarin A [15]	97471101	-
19 ^a^	13.75	C_21_H_18_O_5_	351.1158	351.1227	−6.9	**321.0751**, 305.0864, 293.0823, **279.0685**	6-Hydroxy-3-methoxy-6'',6''-dimethylpyrano[2'',3'':7,8]flavone [21]	64574503	-
20	14.14	C_19_H_16_O_5_	325.1068	325.1071	−0.3	**191.0332**, **176.0125**, 135.0795	2'-Hydroxy-3,4-dimethoxyfurano[4',3':2'',3'']chalcone [37]	6747619	-
21	14.20	C_19_H_16_O_4_	309.1126	309.1121	0.5	**175.0380**, **160.013**	*O*-Methylpongamol [38]	12924865	17877802
22	15.02	C_21_H_18_O_7_	383.1173	383.1125	−0.8	**368.0868**, 353.0663, 321.0431, 307.0594, 293.0445, 279.0647	Pachycarin C [19]	31970912	-
23 ^a^	15.34	C_17_H_10_O_3_	263.0682	263.0703	−2.1	207.0795, 178.0780, **161.0230**, 133.0248, 129.0342, 105.0352	Lanceolatin B [27]	36470090	12225716
24	15.57	C_19_H_16_O_4_	309.1111	309.1121	−1.0	**175.0376**, **160.0171**	Isomer of *O*-Methylpongamol	757311	-
25 ^a^	15.95	C_19_H_14_O_5_	323.0909	323.0914	−0.4	**308.0641**, **293.0442**, 265.0492, 237.0542, 181.0649, **163.0784**, **161.0230**	3',4'-Dimethoxy[2",3":7,8]-furanoflavone [15]	140956269	-
26 ^a^	16.57	C_18_H_12_O_4_	293.0796	293.0808	−1.2	**250.0607**, 182.0709, **161.0231**, 153.0682	Cauliflorin A [18]	85623000	-
27	16.73	C_19_H_16_O_5_	325.1068	325.1071	−0.3	**191.0331**, **176.0125**, 135.0795	Dihydroovalitenin C [39]	7231422	-
28	17.07	C_19_H_16_O_4_	309.1112	309.1121	−0.9	**175.0390**, **160.0111**	Isomer of *O*-Methylpongamol	4454801	-
29 ^a,b^	17.67	C_18_H_12_O_4_	293.0796	293.0808	−1.2	**278.0653**, **277.0495**, 249.0532, 221.0587, 205.0668, 193.0633, **161.0215**	Karanjin [16]	97751879	83932212
30	17.98	C_21_H_18_O_4_	335.1268	335.1278	−1.0	**305.0791**, **203.0344**, 175.0358, 159.0459, 135.0426	Isopongaflavone [40]	897095	11229663
31	18.38	C_19_H_14_O_5_	323.0901	323.0914	−1.3	**307.0588**, 292.0462, 279.0613, **264.0484**,173.0247, **161.0195**, 145.0266	3,6-Dimethoxyfurano-[7,8:2'',3'']falvone [41]	40394167	-
32	19.25	C_23_H_24_O_5_	381.1695	381.1697	−0.2	**247.0951**, **217.1648**, 215.0691, **161.0588**, 159.0810	3'- *O*-Methylpraecansone B [42]	-	4875446
33 ^a^	19.54	C_21_H_20_O_4_	337.1428	337.1434	−0.6	**305.1085**, **233.0792**, 193.0433, 191.0368, 163.0373	7-Methoxy-8-(3''-hydroxy-3''-methyl-1''-butenyl)-flavone [27]	9062891	863827
34 ^a^	20.47	C_22_H_20_O_5_	365.1374	365.1384	−1.0	**335.0896**, **292.0686**, 277.0488, 235.0713, 217.0450, 135.0529	3,6-Dimethoxy-6'',6''-dimethyl-pyrano [2'',3'':7,8]flavone [21]	4240276	-
35	20.63	C_18_H_14_O_3_	279.0996	279.1016	−2.0	205.034, **175.0371**, 149.0230	Ovalitenin A [43]	-	3619252
36	21.29	C_22_H_20_O_5_	365.1382	365.1384	0.6	**335.0899**, **320.0646**, 292.0768, 263.0679, 247.0674, 236.0828	5-Methoxykaranjachromene [44]	1605015	-
37	22.50	C_22_H_22_O_4_	351.1562	351.1591	−2.9	**217.0853**,175.0415, **161.0595**, **147.0450**, 115.0464	4',7-Dimethoxy-8-prenyl-isoflavone [45]	534978	3526648
38 ^a^	23.11	C_20_H_16_O_3_	305.1163	305.1172	−0.9	287.1093, **187.0367**	6'',6''-Dimethylpyrano[2'',3'':7,8]flavone [46]	3297595	56639448
39 ^a^	23.80	C_17_H_10_O_4_	279.0641	279.0652	−1.1	251.0669, **177.0142**, **149.0236**, 121.0246	Pongaglabol [15]	4677607	-
40 ^a^	24.54	C_21_H_18_O_4_	335.1270	335.1278	−0.8	**317.1153**, **305.0808**, 274.0978, **187.0387**, 159.0401, 131.0489	Karanjachromene [15]	75652383	734535
41 ^a^	26.33	C_21_H_22_O_3_	323.1617	323.1642	−2.5	179.0712, **163.0395**, 145.1034,133.0274	Isoderricin A [47]	40392988	-

Notes: **^a^** reported in FC; **^b^** reported in MP; -, not detected.

The HPLC-DAD-ESI-IT-TOF-MS*^n^* method adopted in this study was confirmed to be a powerful method to preliminarily evaluate the ingredients in highly complex Chinese medicine extracts, especially folk medicines and other medicinal plants.

## 3. Experimental

### 3.1. Reagents and Materials

Pachycarin A (**18**), 3',4'-dimethoxy[2",3":7,8]furanoflavone (**25**), karanjin (**29**), pongaglabol (**39**), karanjachromene (**40**) and isoderricin A (**41**) were separated and purified in our laboratory (98%, as determined by HPLC). The chemical structures of the six reference compounds are shown in Figure 3.

Analytical grade methanol and chromatographic grade acetonitrile were purchased from Labscan (Bangkok, Thailand), chromatographic grade formic acid was purchased from Fluka (Buchs, Switzerland). Deionized water was obtained from a Milli-Q water purification system (Millipore, Bedford, MA, USA). 

### 3.2. Plant Material

The sample of *Fordia cauliflora* Hemsl were collected on 12 September 2010, and identified by Professor Shou-Yang Liu (Guangxi TCM University). The root of *Millettia pulchra* (Benth.) Kurz var. *laxior* (Dunn) Z. Wei was collected and identified by professor Ren-Bin Huang (Guangxi Medical University). Voucher specimens are kept in Guangxi Botanic Garden of Medicinal Plant. 

### 3.3. Preparation of Sample Solutions

Representative samples were ground into powder and passed through a 40 mesh sieve. Sample powder (0.2 g) was accurately weighed and transferred into a 50-mL centrifuge tube. Methanol (20 mL) was added and the mixture was sonicated at room temperature for 30 min. The extract was centrifuged at 3,000 rpm for 10 min. The supernatant was filtered with a 0.22 μm filter and injected into the HPLC system. 

### 3.4. HPLC-DAD-ESI-IT-TOF-MS^n^

High performance liquid chromatography with diode array detector and combined with electrospray ionization ion trap time-of-flight multistage mass spectrometry (HPLC–DAD–ESI-IT-TOF-MS^n^) analyses were performed with a Shimadzu LCMS-IT-TOF instrument, which was composed of two LC-20AD pumps, an SIL-20AC autosampler, a CTO-20A column oven, an SPD-M20A PDA detector, a CBM-20A system controller, an ESI ion source, and an IT-TOF mass spectrometer (Shimadzu, Kyoto, Japan). For chromatographic separation, a Dionex Acclaim® PolarAdvantage II C18 LC Column (250 mm × 4.6 mm, 5 μm) was used. The mobile phase consisted of 0.1% formic acid (v/v) (A) and acetonitrile (B) using a gradient program of 50%–58% B in 0–17 min, 58%–70% B in 17–20 min, 70%–85% B in 20–26 min, and 85%–90% B in 26–30 min. The solvent flow rate was 1.0 mL/min, the column temperature was set to 40 °C. PDA detector wavelength: 258 nm. A volume of 25 μL was injected into the HPLC-IT-TOF-MS system.

The conditions of ESI-IT-TOF-MS^n^ analysis were listed below: (1) flow rate: 0.2000 mL/min (split from 1.0000 mL/min HPLC effluent); (2) detection mode: positive ion (PI) and negative ion (NI); (3) mass range: MS *m/z* 100–1,000, MS^2^ and MS^3^, *m/z* 50–1,000; (4) heat block and curved desolvation line temperature: 250 °C; nebulizing nitrogen gas flow: 1.5 L/min; Interface voltage: (+), 4.5 kV; (−), −3.5 kV; detector voltage: 1.70 kV; ion accumulation time: 20 ms; relative collision-induced dissociation energy: 50%; (5) MS^2^ and MS^3^ fragmentation were performed by a data-dependent program; (6) All data were recorded and analyzed by Shimadzu software: LCMS solution Version 3.60, Formula Predictor Version 1.2, and Accurate Mass; (7) a trifluoroacetic acid sodium solution (2.5 mM) was used to calibrate the mass range from 50 to 1,000 Da. 

## 4. Conclusions

In the present study, the HPLC-DAD-IT-TOF-MS^n^ technique was used for rapid identification of multiple constituents in the two folk medicines, Fordiae Cauliflorae Radix and Millettiae Pulchrae Radix. As a result, a total of 41 flavonoids were successfully separated and identified, the chemical characteristics of FC and MP were elucidated respectively, resulting in the characterization of both medicines. The present study, compared with the previous studies, showed differences or improvements as follows: first of all, it is the first report of the use of the HPLC-DAD-IT-TOF-MS^n^ method for detecting the chemical constituents in the folk medicines Fordiae Cauliflorae Radix and Millettiae Pulchrae Radix, and to characterize their chemical constituents in details. Furthermore, according to the interpretation of their mass data obtained from HPLC-DAD-IT-TOF-MS^n^ analysis and also taking into account the data provided by the six reference standards and the established in-house library, a total of 41 constituents were systematically characterized and identified in a single run. 41 flavanoids, including two isoflavones (two known), three pterocarpans (three known), one rotenoid, 10 chalcones (two known), 14 furanoflavones (nine known), seven pyranoflavones (four known), two flavones (one known), and two flavonones (one known) were tentatively identified. This is the first report of 19 of these chemicals from these two medicines. Thirdly, the ^1,3^A^+^ ion resulted from the RDA cleavage of C ring, *m/z* 161.0228 (C_9_H_5_O_3_) was the characteristic fragment ion of furanoflavones, while the RDA cleavage ^1,4^A^+^ fragment, *m/z* 187.0382 (C_11_H_6_O_3_^+^) was the characteristic fragment ion for pyranoflavones; which provided important clues for the identification of major flavonoids. Finally and most importantly, the two ethomedicines could be unambiguously distinguished by the results. The identification results showed that the compounds *O*-methylpongaglabol (**10**), millettocalyxin C (**13**), pongamol (**14**), pinnatin (**17**), pachycarin A (**18**), 6-hydroxy-3-methoxy-6'',6''-dimethyl-pyrano[2'',3'':7,8]flavone (**19**), pachycarin C (**22**), 3',4'-dimethoxy[2",3":7,8]furanoflavone (**25**), cauliflorin A (**26**), 3,6-dimethoxyfurano[7,8:2'',3'']flavone (**31**) and isoderricin A (**41**) can be used to distinguish FC from MP. The results also indicated that the HPLC-DAD-ESI-IT-TOF-MS^n^ technique is rapid and effective for structural characterization of chemical constituents in folk medicines. This work has provided comprehensive information for further quality evaluation and pharmacokinetic studies of FC and MP.

## Supplementary Materials

Supplementary materials can be accessed at: http://www.mdpi.com/1420-3049/18/12/15134/s1.
