# Supplementary material for: Characterization of Flavonoids in the Ethomedicine Fordiae Cauliflorae Radix and Its Adulterant Millettiae Pulchrae Radix by HPLC-DAD-ESI-IT-TOF-MSn
Source: Molecules. 2013 Dec 9;18(12):15134–52. doi: 10.3390/molecules181215134 (PMC6290569; doi:10.3390/molecules181215134)
Supplement: Supplementary file 1 [file molecules-18-15134-s002.pdf]

Supplementary Material for
Characterization of Flavonoids in the Ethomedicine Fordiae Cauliflorae Radix and Its Adulterant Millettiae Pulchrae Radix by HPLC-DAD-ESI-IT-TOF-MS^n

Table 1. Reported chemicals in *Fordia cauliflora* (fc) and *Millettia pulchra* var. *laxior* (mp).

No.	Formula	No. in the Manuscript	Molecular Weight	Chemical Name	fc	mp
1.	$C_{18}H_{12}O_4$	29 [a,b]	292	Karanjin [1]	▲	▲
2.	$C_{19}H_{11}O_5$		322	3-methoxy-2-[3'-methoxyphenyl-4H-furo[2,3-h]-1-benzopyran-4-one [2]	▲	
3.	$C_{18}H_{12}O_4$	10 [a]	2928	O-Methylpongaglabol [2]	▲	
4.	$C_{18}H_{12}O_5$	8 [a]	308	Pongapinnol C [3]	▲	
5.	$C_{18}H_{12}O_4$	17 [a]	292	Pinnatin [3]	▲	
6.	$C_{18}H_{12}O_4$	26 [a]	292	Cauliflorin A [3]	▲	
7.	$C_{15}H_{10}O_4$	3 [a]	254	7,4'-dihydroxyisoflavone [4]	▲	
8.	$C_{16}H_{12}O_4$	12 [a]	268	7-hydroxy-4'-methoxyisoflavone [4]	▲	
9.	$C_{21}H_{18}O_5$	19 [a]	350	6-Hydroxy-3-methoxy-6",6"-dimethylpyrano[2",3":7,8]flavone [4]	▲	
10.	$C_{22}H_{20}O_5$	34 [a]	364	3,6-Dimethoxy-6",6"-dimethylpyrano [2",3":7,8]flavone [4]	▲	
11.	$C_{26}H_{26}O_5$		418	3-methoxy-6-(3-methylbut-2-enyloxy)-6″,6″-dimethylchromeno-(2″,3″:7,8)–flavone [4]	▲	
12.	$C_{21}H_{22}O_3$	41 [a]	322	Isoderricin A [5]	▲	
13.	$C_{25}H_{26}O_3$		374	8,8-dimethyl-2-phenyl-10-prenyl-2, 3-dihydro-8H-pyrano[3, 2-g] chroman-4-one [5]	▲	
14.	$C_{25}H_{28}O_3$		376	7-hydroxy-6, 8-diprenylflavanone [5]	▲	
15.	$C_{20}H_{18}O_5$	11 [a]	338	β,2',5'-trimethoxyfurano [4",5":3',4']-chalcone [6]	▲	
16.	$C_{19}H_{20}O_5$		328	β,2',4',5'-tetramethoxychalcone [6]	▲	
17.	$C_{17}H_{10}O_3$	23 [a]	262	Lanceolatin B [7]	▲	
18.	$C_{21}H_{20}O_4$	33 [a]	336	7-methoxyl-8-(3"-hydroxy-3"-methyl-1"-butenyl)-flavone [7]	▲	
19.	$C_{20}H_{16}O_6$	18 [a]	352	Pachycarin A [8]	▲	
20.	$C_{19}H_{14}O_5$	25 [a]	322	3',4'-dimethoxy (2",3":7,8) furanoflavone [8]	▲	

Table 1. *Cont.*

No.	Formula	No. in the Manuscript	Molecular Weight	Chemical Name	fc	mp
21.	$C_{17}H_{10}O_4$	39 [a]	278	Pongaglabol [8]	▲	
22.	$C_{21}H_{18}O_4$	40 [a]	334	Karanjachromene [8]	▲	
23.	$C_{20}H_{16}O_3$	38 [a]	304	6",6"-Dimethylpyrano[2",3":7,8]flavone [9]	▲	
24.	$C_{18}H_{14}O_4$	14 [b]	294	Pongamol [10]		▲
25.	$C_{17}H_{16}0_3$		268	cis-2, 6-dimethoxyl chalcone [10]		▲
26.	$C_{16}H_{12}O_5$	1 [b]	284	(−)-maackiain [11]		▲
27.	$C_{18}H_{16}O_6$	2 [b]	328	(6*S*, 6a*S*, 11a*R*)-6α-methoxypterocarpin [11]		▲
28.	$C_{17}H_{14}O_5$	7 [b]	298	Pterocarpin [11]		▲
29.	$C_{30}H_{36}O_4$		460	(−)-sophoranone [11]		▲
30.	$C_{18}H_{18}O_5$		314	(6*S*, 6a*S*,11aR)-6α-methoxyhomopterocarpin [11]		▲
31.	$C_{30}H_{36}O_5$		476	(2*S*)5,7,4'-trihydroxy-8,3',5'-triprenylflavanone [11]		▲
32.	$C_{30}H_{36}O_5$		476	(2*R*,3*R*)7,4'-dihydroxy-8,3',5'-triprenyldihydroflavanol [11]		▲
33.	$C_{25}H_{26}O_6$		422	5,7,2',4'-tetrahydroxy-6,3'-diprenylisoflavone [11]		▲
34.	$C_{26}H_{28}O_6$		436	5,7,4'-trihydroxy-2'-methoxy-6,3'-diprenylisoflavone [11]		▲

Note: ▲ reported in this plant; [a] detected in Fordiae Cauliflorae Radix in the manuscript; [b] detected in Millettiae Pulchrae Radix in the manuscript.

References

1. Dai, B.; Cui, C.C.; Dai, X.D.; Xiang, S.F. Chemival constituents of *Fordia cauliflora* (I). *Zhong Cao Yao* **2003**, *34*, 21–22.
2. Dai, X.D.; Yang, D.A.; Dai, B.; Cui, C.C. Chemival constituents of *Fordia cauliflora* (II). *Zhong Cao Yao* **2003**, *34*, 401–402.
3. Dai, B.; Dai, X.D.; Yang, D.A.; Qiu, C.C. Chemival constituents of *Fordia cauliflora* (III). *Zhong Cao Yao* **2003**, *34*, 1063–1065.
4. Liang, Z.Y.; Yang, X.S.; Zhu, H.Y.; Hao, X.J. Two new flavones from *Fordia cauliflora* of Yunnan. *Yao Xue Xue Bao* **2006**, *41*, 533–536.
5. Liang, Z.Y.; Yang, X.S.; Zhu, H.Y.; Hao, X.J. Three Prenylflavanones from *Fordia cauliflora*. *Nat. Prod. Res. Dev.* **2005**, *17*, 592–594.
6. Liang, Z.Y.; Yang, X.S.; Wang, Y.; Hao, X.J.; Sun, Q.Y. Two new chalcones from *Fordia cauliflora*. *Chin. Chem. Lett.* **2010**, *21*, 818–820.
7. Liu, J.L.; Pan, Z.H.; Su, T.; Yan, X.J.; Li, D.P. Chemical constituents from twigs and leaves of *Fordia cauliflora*. *Zhong Cao Yao* **2012**, *43*, 1071–1074.
8. Fan, L.L.; Zhang, Y.Z.; Huang, R.B.; Qin, S.D.; Yi, T.; Xu, F.; Tang, Y.N.; Qu, X.S.; Chen, H.B.; Miao, J.H. Determination of five flavonoids in different parts of *Fordia cauliflora* by ultra performance liquid chromatography/triple-quadrupole mass spectrometry and chemical comparison with the root of *Millettia pulchra* var. *laxior*. *Chem. Cent. J.* **2013**, *7*, 126–134.
9. Ren, L.L. *Study on Bioactivities and the Chemical Components of Alcohol Extract from Fordia Cauliflora Hemsl*; Nanjing University of Technology: Nanjing, China, 2004.
10. Jian, J.; Zhang, S.J.; Qiu, L.; Huang, J.C.; Huang, R.B. Study on chemical constituents of chalcones from *Millettia Pulchra*. *Chin. J. Hosp. Pharm.* **2010**, *30*, 1734–1737.
11. Baruah, P.; Barua, N.C.; Sharma, R.P.; Baruah, J.N.; Kulanthaivel, P.; Herz, W. Flavonoids from *Millettia pulchra*. *Phytochemistry* **1984**, *23*, 443–447.
